# Ethical Aspects of Blood Donors and the Recipients of Their Blood

**DOI:** 10.1155/2012/606753

**Published:** 2011-12-29

**Authors:** P. J. M. van den Burg, K. Magnussen

**Affiliations:** ^1^Sanquin blood Supply, Plesmanlaan 125, 1066 CX Amsterdam, The Netherlands; ^2^Centre for Donor-Haemoglobin and Iron, Blood Bank, Department of Clinical Immunology, sec. 526, Rigshospitalet and Hvidovre Hospital, The Capital Region, 2650 Hvidovre, Denmark

## Abstract

To date medical care is inextricable based on blood donors and blood products. The continuing increase and intensification of tests and guidelines also results in a change in deferral and abnormal test results. Donors and recipients of their blood are faced with this information and are confronted with a kaleidoscope of thoughts and emotions. The discussion with respect to paid versus nonpaid donation is not new, but other aspects are less often discussed. We describe these other aspects for donors and recipients of their blood and hope to open the ethical discussion; if and to what extent we should have limits?

## 1. Introduction

Blood transfusion has developed for about 100 years from “simple” whole blood infusion to advanced targeted therapy with specific parts of donor blood. In the same period enormous innovations were made in production techniques, laboratory techniques, and communication. The field of blood donation and transfusion nowadays plays in a rapidly growing global world with increasing needs for continuous monitoring of new threats and challenges. Simultaneously necessary ethical discussions rise on medical, financial, and political aspects. A number of ethical issues are more often discussed and published, for example, remunerated versus nonremunerated blood donation, and will not be discussed here. In this paper attention is paid towards ethical aspects of donor deferral and donor and recipient information, with focus on the donation of blood, blood products, and stem cells. These topics are less often discussed and published. 

## 2. Donor Deferral, Good Intentions with Unwanted Adverse Effects

For optimal safety of blood donation and transfusion to recipients, guidelines for donor examination are in place. In general donors are voluntary and healthy but may, however, be confronted with deferral. This often unexpected deferral can have several unintended adverse effects as follows.

**Feelings of Rejection**

In general donors feel healthy, have the intention to help a patient, take their time to come to a donor centre, and may not be allowed to donate their blood. A common reason is that the haemoglobin level is just below the lower limit. The donor is informed that, although the haemoglobin level is normal, it is too low do donate. A number of these donors are frustrated and disappointed and leave the donor centre feeling that they are not good enough to help a patient [1, 2]. Most likely this has an impact since it is known that deferrals due to low hemoglobin have a strong effect on return rates of both first-time and repeat donors [3].

**Confrontation with “Old” Diseases**

Donors with a previous history of disorders such as cardiovascular or malignant diseases may have undergone treatment, are asymptomatic, but shall in most cases be deferred. However, these donors generally feel cured, may nearly have forgotten their disease, and are now confronted with a medical deferral. Despite the physician or nurse tries to ease the disappointed donor, a number of donors relive their disease.

**Confrontation with Unrealised Risks**

A number of donor guidelines have a preventive character and are based on enhanced risks of diseases. The risk may be almost negligible while the disease is very serious. In case of deferral with respect to increased risks of v-CJD, for example, cornea transplant or hepatitis B risks in case of endoscopy, the donors are confronted with risks they mostly are not aware of.

**Feelings of Discrimination**

Blood donors belonging to minority groups are prone to feel discriminated upon, when deferred. All who come to donate wants to help, and, when deferred on top of the feeling of rejection, they may also feel discriminated upon. Groups where the incidence of blood-borne infectious disease is higher than that in the background population are deferred to reduce the number of infected blood components from donors who, at the time of donation, were in the window period. Examples of such groups are men who have sex with men (MSM) and couples where one of them come from countries that cause deferral due to risk of contagious and blood-borne infections. Especially the MSM discussion, already started in 1977, is still a matter of debate with many social and ethical aspects [4, 5]. Donors may feel that they, or their partner, are discriminated upon just because of sexual preference or race. In line with this discussion, donors appeal on a supposed right to donate blood that does not exist [6]. It could also be that this reaction to the deferral is actually a result of becoming aware of risks that the donor perhaps repressed or did not realise before! In some countries there have been lawsuits because of this point that interferes with very personal aspects.

## 3. The Problem of Different or Absence of Guidelines

Guidelines can differ, mostly on minor points, between blood establishments and other institutions that collect blood and blood products. Donors can be faced with these differences when different institutions are visited or consulted. We can explain how these differences can exist; however, this may not give a professional impression to donor who asks himself how substantiated our guidelines are. Another point of discussion is not the differences, but the absence of a guideline in specific situations, for example, the Ehlers-Danlos syndrome. The physician should make a decision regarding both donor and recipient safety. The problem occurs when two physicians make different decisions in the same case. We should be aware that this situation degrades the professional position we have.

The reasons for deferral are usually sound, but are not always easy to explain to the blood donors. Mostly the donor is rationally agreeing with the deferral; the personal and emotional experience of this deferral could, however, be rather different. In those situations the donor leaves the blood bank less happy than he came and maybe also worried. The donor may ask “is my intention to donate blood worth the inconvenience.” It is important to be aware of the adverse effects of our rules of deferral already when making the guidelines. Knowing the psychological and practical effects will help us to deal with the problems and enable better assessment regarding the deferrals. Every deferral reduces the probability for retaining the donor [7, 8]. Possible ways of reducing this effect are donor information, education of the staff in deferral rules and donor communication, and to contact the donor after the deferral.

## 4. Information to Donors and Recipients

**Confrontation of Donors with (Unexpected) Information**

Despite donors fill in elaborate questionnaires and test negative in obligatory tests, the recipients of their blood may experience transfusion complications. In certain situations, for example, posttransfusion infections or TRALI, it is warranted to reexamine the donor. The donor is confronted with the knowledge that a recipient could have complaints or disease because of his blood and feels guilty. On the other hand, the donor could have, for example, an infection that was not known and was not discovered during screening of the donor's blood.

**Confrontation of Recipients with (Unexpected) Information**

The recipients are entitled to receive information about the risk of adverse effects to blood transfusion. But after discharge from hospital, adverse effects are hopefully not what the recipient is thinking about. Still, in case of look back due to the risk of a “window donation,” the recipient is, perhaps years after transfusion, confronted with the risk of carrying an infection. Also, before informing the recipient, the physician is troubled with the consideration whether informing the recipient is the best way to go, for example, is case of a short life expectancy. Another striking example is the awareness of risks of transmitting the Creutzfeldt-Jakob disease and later its variant type in relation to Bovine Spongiform Encephalopathy, which resulted in many ethical discussions whether or not to inform recipients [9, 10].

**Influences of Nonanonymous Transfusions**

Donors and recipients, whenever possible, should be anonymous. In case of “related stem cell donation” or patients with very rare blood groups and antibodies against common antigens, this is not always possible. The same is the case in countries where family replacement donations are used. This could have positive effects, but they are by far outbalanced by the negative effects. The donor experiences an emotional pressure that his donation is necessary for the treatment of the patient. As a possible result, the donor could be embarrassed to disclose risk behaviour or medication, both of which could influence the answers in the donor questionnaire. The recipient may not want to be indebted towards a particular donor or the donor may feel that the recipient is now in debt to him. Also the donor will be aware of the patient's condition and could feel guilty if the condition is aggravated.

**Confrontation with (Unforeseen) Information**

These issues with respect to test results or risks for diseases are applicable to the field of, mainly unrelated, stem cell transplantations. After transplantation the donor can develop a disease that could also have been “transmitted” by the stem cells. Also the recipients can develop a disease that is likely or could have originated form the transplanted cells. In what case should we inform the donor or the recipient? Do therapeutic options play a role or is it relevant for genetic counselling of relatives?

**The Scope of Informed Consents**

All the possible risks of blood, blood products, or stem cell donation can be included in the information to donors and recipients as part of the informed consent [10, 11]. However, do we serve the needs of donors and recipients when we strive to give all information? Do we want to face donors and recipients of their blood to all conceivable results and adverse effects? Do the donors and the recipients of their blood want to be informed about all the information that physicians, or lawyers, want to give? Independent of these questions we should also realise that donors and recipients do not read or understand all information and also are selective for the information provided [10–12].

**Confrontation with Test Results**

An increasing number of tests are developed to improve the safety and efficacy of blood transfusion. And information to the donor, about the tests that are done, is mandatory. In these informed consents attention should be paid to the kind of tests and information about, possible unexpected, test results [13]. Whenever a test is positive, the donor is retested and informed when confirmed positive. Clearly, confirmed positive test results for infectious diseases have great influence. However, (temporary) deferral because of seemingly unimportant false-positive test results is often misunderstood and has negative psychosocial effects [14–16]. Beside results from infectious disease tests, donors can also be confronted with other tests that interfere with donorship such as positive DAT results or cold agglutinins that hinders leucofiltration. How specific and reliable must a test result be to inform donors about them? To what extent must donors be protected for results that have no consequences at that moment in time, and shall mainly be an emotional burden?

## 5. Information and Advices

Information and advice about risks and adverse effects are important, also, because in some cases knowledge may reduce the risk. In the case of needle injury, correct phlebotomy technique together with compression and only light use of the donation arm after the donation will reduce the risk of needle injury [17].

Iron deficiency is common among the general population and even more common among blood donors. Iron deficiency may result in overt anemia or a lower haemoglobin than what would have been the normal haemoglobin level for a particular person. Iron deficiency in itself also have effects, like muscle fatigue, reduced endurance, and perhaps also impaired cognitive skills, and also means low iron stores in case of pregnancy or blood loss [18–20].

There are striking differences with respect to the fact if we advise, treat, or only wait longer for the next donation. Some blood centres do not test for iron deficiency or only do so when the haemoglobin level is below the limit for donation, but still more develop protocols to avoid the blood donors from developing iron deficiency and to diagnose the iron deficiency before the donor develops anaemia. In some institutions it is common to give dietary advices; other institutions advise or subscribe iron supplementation; other increase the interval between donations or permanently defer the donor. To what extent do we want to interfere with life style or treat donors who are healthy? Do we accept adverse effects of ironsupplementation in healthy asymptomatic donors to increase with these efforts their haemoglobin, which is subsequently donated in the following donation? Should we accept the adverse effects of iron deficiency that is caused by donations, without acting on the current knowledge? 

## 6. Conclusion

In this paper we describe a number of ethical issues that could play a role in donors and recipients of blood, blood products, and stem cells. We try to focus on these issues to get attention and awareness that these items are of influence. In counselling donors it is important that we not only send the message we want to give but also receive the verbal and nonverbal information of donors. Only by receiving that information of the donor, we are able to give adequate information. Most likely, we shall be confronted with other ethical aspects in the future. Medical and ethical aspects are like a snapshot and will need renewal regularly.
